# Worlds apart or two sides of the same coin? Attitudes, meanings, and motives of potential oocyte and sperm donors in Austria

**DOI:** 10.1007/s10815-019-01683-8

**Published:** 2020-01-08

**Authors:** M. Flatscher-Thöni, B. Böttcher, W. Geser, A. Lampe, G. Werner-Felmayer, C. Voithofer, C. Schusterschitz

**Affiliations:** 1 Department of Public Health, Health Services Research and Health Technology Assessment, UMIT – University for Health Sciences, Medical Informatics and Technology, 6060 Hall in Tyrol, Austria; 2 Department of Gynecological Endocrinology and Reproductive Medicine, Medical University of Innsbruck, Anichstrasse 35, 6020 Innsbruck, Austria; 3 Institute of Psychology, University of Innsbruck, 6020 Innsbruck, Austria; 4 University Clinic of Medical Psychology, Medical University of Innsbruck, 6020 Innsbruck, Austria; 5 Division of Biological Chemistry, Biocentre, Medical University of Innsbruck, bioethics network education, 6020 Innsbruck, Austria; 6 Department of Civil Law, University of Innsbruck, 6020 Innsbruck, Austria; 7 Department of Psychology and Medical Sciences, UMIT – University for Health Sciences, Medical Informatics and Technology, 6060 Hall in Tyrol, Austria

**Keywords:** Oocyte donation, Sperm donation, Altruism, Attitude, Genetic link, Motives

## Abstract

**Purpose:**

Gamete donors and recipients of such donations have been explored by previous studies, which mostly focus on post-donation scenarios. Our study analyses the general willingness to donate oocytes or sperm and focuses on differences between potential female and male donors in attitudes, meanings, and motives in a pre-donation setting.

**Methods:**

An electronic survey (*n* = 555 students) was used in this anonymous observational study. To enable comparisons between men and women regarding their attitudes, meanings, and motives and their willingness to donate gametes, we designed two separate questionnaires.

**Results:**

The sample was divided into three groups based on the willingness to donate: potential donors (*n* = 133; women: 48.1%, men: 51.9%); doubtful donors (*n* = 207; women: 75.8%, men: 24.2%); and non-donors (*n* = 215; women: 68.3%, men: 31.7%). The group of potential male donors (39.2%) was significantly larger than the group of potential female donors (16.9%). Significant differences regarding altruism, the meaning of one’s self-worth, and passing on the own genes were found between doubtful and potential donors. Potential donors attached less value to altruism but more value to the enhancement of one’s self-worth and passing on one’s genes than doubtful donors. The motive of passing on one’s genes and altruistic motives were more important to men than to women.

**Conclusion:**

This study helps to create a better understanding of potential donors in the existing donation framework and supports the evaluation of the given regimes in the context of designing an improved framework.

## Introduction

Reproductive medicine is a dynamic field of medical science and is therefore also influenced by societal and political changes. At present, legal regulations on assisted reproductive technologies (ART) have not yet been harmonized within Europe. Therefore, the scope of the existing regulations varies, does not reflect a certain standard, and can be summarized as a legal mosaic [1].

The Austrian law on artificial procreation (The Artificial Procreation Act = “Fortpflanzungsmedizingesetz”, Federal Law Gazette 275/1992) entered into force in 1992 and underwent fundamental changes in the year 2015, when the formerly quite restrictive regime was liberalized [2]. Since then, oocyte donation has been permitted for heterosexual couples but not for single women. Recruitment and reimbursement of gamete donors are not approved. Sperm donation has been allowed since 1992 for insemination only but is now also authorized for in vitro fertilizations and for homosexual couples.

The new legal situation stresses the fact that ART is a dynamic field of law that reacts to society’s changing value system as well as new developments in medicine and technology. However, little is known about the (legal) needs and the perception of the involved interest groups, e.g. medical institutions/physicians, children to be conceived, and gamete providers, although this information is necessary to appropriately evaluate the legal framework of ART.

Focusing on the interest group of gamete providers seems of special interest for countries such as Austria, where the legal system has recently accepted fundamental changes to the donation system. Due to this change, a sound understanding of the social and psychological factors determining intentions to donate gametes is not only important for clinics, policymakers, and recruitment campaigns but also for lawmakers [3]. People choose to provide gametes to others for a variety of reasons, including compensation or the desire to help [4].

Only a limited number of (European) studies actually focus on these aspects of gamete provision. These studies can generally be divided into post-donation (focusing on actual donors) and pre-donation (focusing on potential donors) settings. The characteristics and motives of oocyte and sperm donors, egg share donors, and recipients have been explored previously [3, 5–9], mainly in populations where oocyte and sperm donation have been permitted for several years. Therefore, most studies have used post-donation questionnaires or interviews, possibly leading to the bias that donors with positive experiences and attitudes tend to participate [9]. The main issues of these studies include motivation stemming from financial compensation and the risk of donor exploitation vs. altruistic donation; anonymous donation and disclosure to the offspring; as well as the health risks to potential oocyte donors and personal experiences. In a systematic review of actual sperm donors, four different types of motives were identified: altruism, financial compensation, procreation of genetic fatherhood, and the status of their own fertility [8].

One study in particular focused on the characterization of potential oocyte donors: Svanberg et al. performed a questionnaire study in Sweden in 2003, exploring factors influencing women’s willingness to donate [10, 11]. Additionally, Svanberg et al. [10] analyse donors’ motivation and ambivalence before the donation of gametes. Their results show that, in general, motives to donate gametes are mainly altruistic. Men and women differ in their views of donating gametes, and sperm donors have a higher degree of ambivalence towards donation than do oocyte donors. In contrast, Nüssli et al. [12] solely focused on young women in Switzerland and asked under which conditions they would be willing to provide their oocytes. The respective results show that approximately 60% of the women are generally willing to donate their oocytes and that altruism is their main motivation.

To our knowledge, this is the first pre-donation study of potential gamete donors in Austria. By focusing on men as well as on women, we aimed to analyse differences between the male and female willingness to donate. We are fully aware that oocyte donation and sperm donation cannot be compared regarding the physical burden of the donation procedures. By exploring a societal group of possible gamete donors, we wanted to establish a better understanding of the differences in general attitudes towards gamete donation as well as in meanings of gametes and motives for donation. Moreover, we were interested in circumstances influencing potential donation by men and women. This topic is particularly relevant against the background of the recent amendment of the Austrian Artificial Procreation Act.

Our specific aims were to explore the following research questions:

- Do men and women differ in their willingness to donate gametes?
- Do potential, doubtful, and non-donors differ in their attitudes towards gamete donation and their meanings of gametes, and are there sex differences within the donor groups?
- Do potential and doubtful donors differ regarding their motives for gamete donation, and are there sex differences within these donor groups?
- What special circumstances would make gamete donation among the male and female donor groups more likely?

## Material and methods

### Participants and procedures

The research population consisted of approximately 32,000 students from three different Austrian universities. The students received an email invitation to participate in this online survey. The email included a short study description and a link leading directly to the survey. Instructions on how to fill out the questionnaire were given online after participants had read and confirmed agreement on the informed consent form. Hence, the participation was voluntary and anonymous. The questionnaire could be completed in 20 min.

Between April and June 2017, 922 students participated in the online study. Three hundred sixty-five students have not completed the questionnaire, while 555 have fully answered the questionnaire. We do not find significant gender differences for the ones who did not finish. For the analysis at hand, we decided to only use completed datasets.

All procedures performed in the study were in accordance with the ethical standards of the institutional ethics committees and with the 1964 Helsinki Declaration and its later amendments. The Board of Ethical Questions in Science of the University of Innsbruck approved the study design (Certificate of good standing 02_2017), as did the Research Committee for Scientific and Ethical Questions (RCSEQ) at UMIT – University for Health Sciences, Medical Informatics and Technology (Certificate of good standing 2060).

### Questionnaires

Two questionnaires, a female and a male version, were designed based on international literature and an internal review process of an interdisciplinary research team, consisting of reproductive physicians, jurists, psychologists, ethicists, and psychiatrists, two of each profession. Based on the outcome of a pretest among 50 students, changes were made concerning the phrasing of some vague wordings.

Both versions of the questionnaire generally consist of the same 44 items, intending to reveal respondents’ attitudes, meanings, and motives, as well as the willingness to donate. The only difference is that items in the questionnaire for female respondents exclusively refer to oocyte donation, while the items in the questionnaire for male respondents exclusively focus on sperm donation. This difference enables the analysis of gender differences. Example items measuring motives and perceptions of gamete donation, as well as meanings ascribed to gametes, are given below in connection with the scale descriptions.

If not otherwise stated, participants responded to all measures presented below on a six-point Likert rating scale (1 “not true at all”, 6 “absolutely true”). To ensure that high item scores consistently indicate a high degree of agreement, some items had to be recoded.

In detail, the first part of the questionnaires focused on the willingness to donate oocytes or sperm. In line with Svanberg et al. [10], we assessed the willingness to donate oocytes or sperm with the following items: Would you consider donating oocytes (female questionnaire)/sperm (male questionnaire) at some point in the future? This question had to be answered by choosing from the response options “yes”, “maybe”, and “no”.

Part two of the questionnaire measured the general attitudes towards oocyte/sperm donation, which were assessed with the five items formulated by Svanberg et al. [10]. Example items of this scale are “Oocytes (female questionnaire)/ Sperm (male questionnaire) donation is a good way to help childless couples” and “If you cannot have children on your own, you should not have any”. Cronbach’s alpha for this scale was α = .83. To reveal participants’ meanings of their oocytes and sperm, we formulated six items. Exploratory factor analyses revealed two separate factors, which we labelled personal vs. factual meaning of gametes. The subscale “personal meaning of gametes” consists of 4 items (α = .74), and the subscale “factual meaning” consists of 2 items (r = .34, *p* ≤ .00). An example item for the factual meaning factor is “I perceive my oocytes/sperm as comparable to blood or plasma”. Example items for the personal meaning factor are “I perceive my oocytes/sperm as part of myself” and “I perceive my oocytes/sperm as foundation of future life”.

In part three, our questionnaire also asked for respondents’ motives, based on the literature and previous works [6, 9, 10, 13–17]. In detail, participants indicated the role of (1) altruism, (2) passing on one’s genes, (3) financial reimbursement, and (4) self-enhancement regarding their willingness to donate. Each motive was assessed with a single item. Participants additionally provided information on whether the type of relationship they have with the recipients would influence their willingness to donate. Only participants who were willing to make their oocytes/sperm available for assisted reproduction were asked to respond to the motive items and to those referring to the nature of the relationship with the recipients.

Part four included twelve items focussing on different aspects of the donation procedure itself that could positively influence the willingness to donate. These items were formulated with reference to Svanberg et al. [10]. Examples for such factors and thus for the response options provided are “you could talk to a woman/man who has already donated oocytes/sperm” and “you would know the recipients, you would already have own children, or you would have been asked to donate at a medical check-up”.

Finally, participants had the option to add their own comments as free text in a qualitative response section.

The questionnaire ended with questions about socio-demographic features of the students (age, relationship status, sexual orientation, religious beliefs, etc.).

Based on the item concerning participants’ willingness to donate oocytes/sperm at some point in the future, the study sample was subdivided into three groups: potential donors (*n* = 133; 24%; women: 48.1%, men: 51.9%) who could imagine donating oocytes/sperm at some point in the future; doubtful donors (*n* = 207; 37.3%; women: 75.8%, man: 24.2%) who were unsure in this regard; and non-donors (*n* = 215; 38.7%; women: 68.3%, man: 31.7%) who would not donate oocytes/sperm.

### Data analysis

Where not otherwise stated, two-way analyses of variance (ANOVA) and subsequent one-way analyses of variance with post hoc tests (Scheffé) were used to analyse differences among the three donor groups and sex differences in the variables of interest. To reveal significant differences in the distribution of the three donor groups (potential/ doubtful/ and non-donors) over our study variables, we used chi-square tests. *p* values < .05 (two-sided) were considered statistically significant.

All statistical analyses were performed with IBM SPSS (v22.0).

## Results

### Socio-demographic characteristics and donor groups

Of the participating students (*n* = 555), 68% were female and 32% were male. More than half of the students (58.5%) lived in a stable relationship, while the rest were single (41.5%) at the time of the survey. The vast majority considered themselves heterosexual (90.1%) and had no children yet (92.2%). A total of 61.7% belonged to a religious community, whereas 23.3% were without confession. The sample included students from a wide variety of fields of study, namely, humanities (4.1%), natural sciences (5.6%), economic sciences (4.3%), educational sciences (4.7%), engineering sciences (1.3%), medicine (62.2%), social sciences (12.2%), and law (5.5%). For absolute numbers of all socio-demographic variables, see Table 1.

**Table 1:** Socio-demographic characteristics: frequency distributions and means

*Overall participants*	***Female***	***Male***
	379	176
*Age*	23.38 (SD 3.82)	25.64 (SD 6.55)
*Nationality:*		
*Austrian*	226	113
*German*	77	34
*Italian*	61	24
*Other*	15	5
*Religious affiliation*	273	123
*No religious affiliation*	84	44
*Relationship:*		
*Single*	154	74
*In relationship*	221	99
*Sexual orientation:*		
*Heterosexual*	344	154
*Homosexual*	7	11
*Bisexual*	20	10
*Own children*	1	1.29
*Non-donors*	157	50
*Potential donors*	64	69
*Doubtful donors*	158	57

*SD* standard deviation

The distribution of male and female students over the three donor groups (potential/ doubtful/ and non-donors) showed that the group of female potential donors (16.9%) was significantly smaller than that of male potential donors (39.2%), while the groups of female non- (41.4%) and doubtful donors (41.7%) were significantly larger than the groups of male non- (28.4%) and doubtful donors (32.4%) (chi^2^ = 33.13; df = 2; *p* < .00).

Individuals with religious affiliation (21.3% potential donors, 38.6% non-donors, 40.1% doubtful donors) were less willing to donate oocytes/sperm than those without religious affiliation (32% potential donors, 30.5% non-donors, 37.5%) (chi^2^ = 6.23; df = 2, *p* = .04).

Relationship status had no influence on the willingness to donate (chi^2^ = 1.07; df = 2, *p* = .56).

### General attitudes towards gamete donation

To analyse sex differences and differences between the three donor groups regarding general attitudes towards oocyte and sperm donation, respectively, two-way analyses of variance were conducted. While findings demonstrate differences among the three donor groups (F (2,549) = 68.08, *p* < .00), they reveal no sex differences (F(1,549) = 1.13, *p* = .29) and no interaction effects (F(2,549) = 1.97, *p* = .14). Post hoc tests (Scheffé) show that non-donors differ significantly from potential donors and doubtful donors. Mean differences in Table 2 imply that non-donors attitudes towards gamete donation are significantly less positive than those of potential and doubtful donors. In detail, non-donors express lower agreements to statements like “Oocyte/Sperm donation is a good way to help a childless couple” and higher agreements to statements like “If you cannot have children of your own, you should not have any”.

**Table 2:** General attitudes towards gamete donation and meaning ascribed to one’s own gametes

		Potential donors			Non-donors			Doubtful donors			Total	
		Women	Men	Total	Women	Men	Total	Women	Men	Total	Women	Men
General attitude	M	5.28	5.28	5.28^a^	4.14	4.42	4.21^c^	5.02	4.98	5.01^b^	4.70	0.91
	SD	0.45	0.43	0.44	1.03	1.08	1.04	0.56	0.54	0.56	4.94	0.78
Personal meaning of gametes	M	4.71^b^	4.68	4.69	5.33^a^	4.64	5.16	4.94^a^	4.77	4.90	5.06	4.70
	SD	0.82	0.90	0.86	0.60	1.03	0.78	0.77	0.96	0.83	0.75	0.95
Factual meaning of gametes	M	3.57^a^	3.55	3.56	2.32^c^	3.24	2.54	3.02^b^	3.32	3.09	2.82	339
	SD	0.97	1.20	1.09	1.06	1.29	1.19	1.09	1.23	1.14	1.16	1.24

*M* mean, *SD* standard deviation, *a, b, c* different letters (a, b, c) indicate significant (*p* < .05) mean differences (in the Scheffe post hoc test) between the donor groups and between men and women

### Meanings of one’s own gametes

Differences between the three donor groups and sex differences were again tested with analyses of variance and subsequent post hoc tests (Scheffé). Mean values and results of the post hoc tests are summarized in Table 2.

For both dimensions, personal and factual meaning of gametes, we identified highly significant differences among the three donor groups (dimension personal meaning: F_(2,549)_ = 4.55, *p* = .01; dimension factual meaning: F_(2,549)_ = 17.43, *p* = .00) and highly significant sex differences (dimension personal meaning: F_(1,549)_ = 15.59, *p* = .009; dimension factual meaning: F_(1,549)_ = 14.43, *p* = .00) (Table 2). In addition, analyses of variance demonstrate highly significant interaction effects between sex and the affiliation to one of the three donor groups (dimension personal meaning: F_(2,549)_ = 6.92, *p* = .00; dimension factual meaning: F_(2,549)_ = 6.66, *p* = .00).

One-way analyses of variance reveal that the personal meaning men ascribe to their sperm is comparable in all three male donor groups (F_(2,173)_ = .30, *p* = .75). In contrast, the personal meaning that women ascribe to their oocytes is significantly different among the three female donor groups (F_(2,376)_ = 20.49, *p* = .00). In detail, compared with female doubtful- and female non-donors, female potential donors ascribe the lowest personal meaning to their oocytes. The results further demonstrate that for men, the factual meaning of their own sperm is comparable among all three male donor groups (F_(2,173)_ = 1.05, *p* = .35). In contrast one-way ANOVA tests show for all three female donor groups highly significant differences (F_(2,376)_ = 36.46, *p* = .00) regarding the factual meaning of their own gametes. Significant mean differences in Table 2 demonstrate that the factual meaning of their own gametes is lower in the female non-donor group than in female potential donors and female doubtful donors. Moreover, potential donors also perceive their oocytes significantly more factual than doubtful donors. Female non-donors can be said to view their oocytes more strongly than any other female or male donor group as “part of themselves”, “a basis for future life”, or “part of their body”. This conclusion is based on their lowest mean value in the dimension factual meaning and their highest mean value in the dimension personal meaning, together with the significant differences observed between the three female donor groups (see Table 2). At the same time, their agreement to statements like “I consider my oocytes as comparable with blood/ plasma” is lowest.

### Motives for gamete donation

Because motives were assessed solely among study participants falling into the group of doubtful and potential donors, only the motives of these two donor groups were compared.

Mean values of potential and doubtful donors and separate mean values for male and female potential and doubtful donors, together with the significant mean differences, are shown in Table 3.

**Table 3:** Motives for gamete donation

		Potential donors			Doubtful donors			Total	
		Women	Men	Total	Women	Men	Total	Women	Men
For altruistic reasons	M	2.31	2.72	2.53^b^	2.74	2.98	2.80^a^	2.59	2.82^a^
	SD	1.17	1.16	1.18	1.14	1.49	1.24	1.16	1.30
To pass on my genes	M	2.52	3.49	3.02^a^	2.38	2.77	2.48^b^	2.43	3.21^a^
	SD	1.41	1.70	1.64	1.28	1.51	1.36	1.33	1.66
Because of a positive impact on my self-esteem	M	2.67	2.39	2.53^a^	2.34	2.02	2.26^b^	2.46^a^	2.25
	SD	1.50	1.32	1.41	1.26	1.20	1.25	1.36	1.28
Only with financial reimbursement	M	2.73	2.96	2.85	2.76	2.65	2.73	2.75	2.84
	SD	1.52	1.65	1.59	1.41	1.73	1.50	1.45	1.68
Only to family members	M	1.88	1.59	1.73^b^	2.32	1.74	2.16^a^	2.16^a^	1.65
	SD	0.93	0.69	0.83	1.23	0.82	1.16	1.15	0.74
Only to anonymous persons	M	3.11	3.30	3.21	3.26	3.33	3.28	3.21	3.31
	SD	1.68	1.74	1.71	1.49	1.64	1.53	1.56	1.70
Only to friends	M	2.09	2.01	2.05^b^	2.46	2.26	2.41^a^	2.33	2.11
	SD	1.11	1.10	1.10	1.24	1.29	1.26	1.21	1.18

*M* mean, *SD* standard deviation, *a, b, c* different letters (a, b, c) indicate significant (*p* < .05) mean differences (in the Scheffe post hoc test) between the donor groups and between men and women

Results of two-way analyses of variance provide evidence for sex differences and/or differences between doubtful and potential donors in motives for gamete donation, but not for interaction effects.

Doubtful donors differ significantly from potential donors in the role that altruism (F_(1,289)_ = 5.04, *p* = .03), passing on one’s genes (F_(1,289)_ = 5.72, *p* = .02), and enhancing one’s self-worth (F_(1,289)_ = 4.5, *p* = .04) play with respect to the willingness to donate (Table 3). Altruism is more important to doubtful than to potential donors, while passing on one’s genes and enhancing one’s self-worth are more important to potential than to doubtful donors. Beyond that, altruism (F_(1,289)_ = 4.74, *p* = .03) and the motive of passing on one’s genes (F_(1,289)_ = 14.32, *p* = .00) are more relevant to men than to women. For the self-enhancement motive, we found no significant sex differences (F_(1,289)_ = 3.31, *p* = .07).

In regard to the closeness of the recipient, it seems more important to doubtful than to potential donors that donor recipients are either family members (F_(1,289)_ = 5.60, *p* = .02) or loved ones (F_(1,289)_ = 4.23, *p* = .041). Doubtful and potential donors assess donations to unknown persons equally, based on our non-significant results (F_(1,289)_ = .19, *p* = .66). The results additionally reveal that it is more important to women than to men that recipients of their donation are family members F_(1,289)_ = 11.661, *p* = .001). Regarding a donation only to loved ones (F_(1,289)_ = .93, *p* = .34) or only to unknown persons (F_(1,289)_ = .40, *p* = .53), no sex differences were observed.

### Aspects making gamete donation more likely

Finally, we were also interested in relevant circumstances of the donation procedure making gamete donation more likely. Again, two-way analyses of variance with subsequent Scheffé tests were conducted. Results of the Scheffé tests together with the relevant means and standard deviations are presented in Table 4.

**Table 4:** Circumstances making gamete donation more likely

		Potential donors			Non-donors			Doubtful donors			Total	
		Women	Men	Total	Women	Men	Total	Women	Men	Total	Women	Men
Financial compensation	M	3.53	4.00	3.77^a^	2.25	2.82	2.39^b^	3.56	3.72	3.60^a^	3.01	3.59^a^
	SD	1.79	1.66	1.73	1.51	1.60	1.54	1.50	1.76	1.57	1.68	1.74
Close distance to place of residence	M	4.06	3.96	4.01^a^	2.57	2.74	2.61^b^	3.96	4.16	4.01^a^	3.40	3.68
	SD	1.51	1.55	1.53	1.60	1.63	1.60	1.43	1.46	1.44	1.66	1.65
Counselling	M	5.50	4.80	5.14^a^	3.91	3.96	3.92^b^	5.29	4.82	5.17^a^	4.75^a^	4.57
	SD	0.76	1.27	1.11	1.78	1.68	1.75	0.91	1.12	0.99	1.50	1.40
Exchange with ex-donors	M	4.75	3.23	3.96^a^	3.80	2.90	3.58^b^	4.87	3.39	4.47^a^	4.40^a^	3.19
	SD	1.33	1.48	1.60	1.67	1.72	1.72	1.26	1.51	1.48	1.54	1.56
Knowing the recipients	M	3.63	3.25	3.43	3.62	2.74	3.41	3.66	3.19	3.54	3.64^a^	3.10
	SD	1.74	1.58	1.66	1.65	1.71	1.71	1.44	1.62	1.50	1.58	1.64
Own children	M	3.45	2.94	3.19^b^	3.50	2.90	3.35^b^	3.92	3.33	3.77^a^	3.67^a^	3.07
	SD	1.75	1.54	1.66	1.75	1.50	1.71	1.54	1.50	1.55	1.67	1.53
Asked at medical check-up	M	4.27	3.84	4.05^a^	2.20	2.36	2.24^c^	3.66	3.47	3.61^b^	3.16	3.30
	SD	1.31	1.46	1.40	1.25	1.38	1.28	1.16	1.34	1.21	1.48	1.52
Psychological aspects of infertility	M	4.02	4.06	4.04^b^	3.52	3.32	3.47^c^	4.50	4.32	4.45^a^	4.01	3.93
	SD	1.64	1.61	1.62	1.47	1.62	1.51	1.26	1.28	1.26	1.48	1.55
Shorter duration of treatment (oocyte retrieval)	M	4.42		4.42^a^	3.03		3.03^b^	4.54		4.54^a^		
	SD	1.24		1.24	1.60		1.60	1.27		1.27		
Private anonymous setting	M	3.73	4.43	4.10^a^	2.99	4.10	3.26^b^	4.26	4.75	4.39^a^	3.64	4.44^a^
	SD	1.65	1.44	1.58	1.66	1.71	1.74	1.38	1.31	1.38	1.65	1.50
Information about well-being of offspring	M	3.44	3.54	3.49	3.42	3.56	3.45	3.75	3.47	3.67	3.56	3.53
	SD	1.65	1.54	1.59	1.67	1.62	1.65	1.47	1.67	1.53	1.59	1.60

*M* mean, *SD* standard deviation, *a, b, c* different letters (a, b, c) indicate significant (*p* < .05) mean differences (in the Scheffe post hoc test) between the donor groups and between men and women

The three donor groups evaluate precise circumstances of the donation procedure significantly differently with regard to their positive influence on the willingness to donate. Non-donors turn out as least likely to be positively influenced by specific circumstances of the donation procedure. This applies to all circumstances of the donation procedure for which significant group differences were identified (see Table 4). Doubtful donors seem more likely to donate if they already have children on their own, but this is not the case for potential donors and non-donors (F_(2,549)_ = 3.77; *p* = .03). Female non-donors are the least likely to be positively influenced by a short treatment period before oocyte retrieval.

In regard to sex differences in the evaluation of specific circumstances of the donation procedure, the results suggest that receiving financial compensation (F_(1,289)_ = 6.996, *p* = .01) and carrying out the procedure in an anonymous setting (F_(1,289)_ = 28.37, *p* = .00) would make men more likely to donate. In contrast, women would be more likely to donate if they received in-depth counselling (F_(1,289)_ = 8.779, *p* = .003), if they could talk to actual female donors (F_(1,289)_ = 85.11, *p* = .00), if they knew the couple to whom they donated their oocytes (F_(1,289)_ = 14.55, *p* = .00), and if they already had children of their own (F_(1,289)_ = 13.7, *p* = .00).

## Discussion

### Willingness to donate and motives for gamete donation

In our cohort, 24% were willing to donate: 39% of the male participants and 17% of the female participants. Interestingly, comparably the same percentage of potential female donors was identified by Svanberg et al. [10, 11] shortly after the legalization of oocyte donation in Sweden in 2003.

Our study reveals that doubtful donors differ significantly from potential donors in the role that altruism, passing on one’s genes, and enhancing one’s self-esteem play with respect to the willingness to donate. Hence, altruism is more important to doubtful than to potential donors, while passing on one’s genes and enhancing one’s self-worth are more important to potential than to doubtful donors. Beyond that, our results show that altruism and the motive of passing on one’s genes seem more relevant to men than to women. Although we are not aware of studies dealing with these specific gender discrepancies with respect to motives for gamete donation, our findings are in line with reported results for sperm donation. Cook and Golombok [13] concluded that altruistic motives were important in the decision to donate sperm, and studies performed in Sweden and Australia pointed at the relevance of altruistic motivation for male donors [14, 15]. Additionally, the importance of the motive of passing on one’s genes, meaning the desire to procreate, was also revealed by Riggs and Russell [16] who studied the motivation of sperm donors in four different countries.

### General attitude

In our study, donation was seen as a way to help others, indicating a positive attitude towards gamete donation and not favouring adoption or remaining childless instead. In an epidemiological study in 11 countries, purely altruistic motives were found in 47.8% of oocyte donors [1]. Altruistic oocyte donors are influenced by their personal and emotional relationship to the recipient, their own motherhood, and their empathy [17]. In Austria, financial compensation and recruitment of donors are not legal. Therefore, recipients may depend on relatives or friends as oocyte donors. A close relationship has been identified as the main motive, especially between sisters [18]. However, restricting donations between family members has been discussed before [19]. This close relationship between the recipient and donor implies a high risk of personal emotional involvement and even higher pressure to have a successful outcome. A failure can have a significant negative impact on both parties [17]. Donors might feel an obligation to repeat the procedure if the outcome is unsuccessful [20]. On the other hand, positive changes in the relationship between recipient and known donor have also been found in previous studies [17, 21, 22]. In our setting, a psychological state of dependence can develop, similar to the state described after transplantation of a kidney or liver between relatives [23, 24].

In our cohort and in accordance with previous research [17], no association between the willingness to donate and religious beliefs was found.

### Special circumstances

We found that participants were more likely to donate if they were offered financial reimbursement. We suppose that this aspect plays a major role in our cohort, as we questioned students who were not yet earning money. Obviously, it is easier to have purely altruistic motivations when one’s own income is secure. In a previous study analysing socio-demographic characteristics of oocyte donors, it was found that the higher the level of education, the more altruistic the donor was [1]. Previous research showed that a combination of altruistic and financial motives is very common in donors [1, 3]. The presence of financial reimbursement did not mean that these donors were mainly motivated by money. Nevertheless, it was shown that the financial motivation of oocyte donors was greater when more money was offered [25]. Donor characteristics with regard to age, education, and psychological profile remained the same regardless of the amount of compensation [26]. The appeal of financial reimbursement may not lead to ignoring the health risks of oocyte donation. Although our results point at the impact of financial reimbursement, it is necessary to state that the Austrian law on artificial procreation does not allow any provision of gametes in return for payments. Therefore, thorough counselling is of major importance.

Health risks play a minor role in sperm donation; additionally, the effort of donation is less than for oocyte donation. Consequently, one could assume that women would be more likely than men to expect a financial reimbursement for their donation. In contrast, in the present study, significantly more men favour financial compensation than women. In a previous study, 62% of sperm donors would not have donated if they had not received payment [13].

According to our results, thorough counselling about the procedure, its consequences, and the psychological impact on the infertile couple would encourage potential donors to donate. This finding is supported by a previous study showing that over 75% of donors were motivated by their personal knowledge of the psychological burden of childless couples [17]. This motivation also applies to sperm donors [7]. The need for counselling and informed consent should be self-evident in medical practice, especially in the context of procedures with more than one party involved and of sensitive issues such as reproduction. A post-donation questionnaire study showed limited knowledge about possible side effects and long-term consequences of oocyte donation [27]. Furthermore, the relationship between the donor and healthcare provider remains a challenge [28]. Donors seem to be afraid that they are not sufficiently informed about health risks and psychological consequences, as they might be seen only as egg producers, especially if they have signed a contract and received reimbursement [28]. Positive donation experiences are reported by donors who felt that they were treated with respect and that their contribution was appreciated [18].

According to a recent meta-analysis, known oocyte donors tend to be over 30 years old, married, heterosexual, and usually a sister or close friend of the recipient [5, 9]. Participants stated that they would be more likely to donate if they had own children. In our student cohort, the percentage of men and women with children was very low. Thus, it seems that participants want to already have their own offspring before they donate to others. For sperm donors, this finding is in contrast to the literature, as the typical sperm donor is single, childless, and highly educated [20].

### The meaning of a child and the genetic link

In our study, non-donors stated that the genetic link to the offspring was of major importance to them. Non-donors also regard children as one purpose of life. From an altruistic point of view, one could assume that these participants would be especially inclined to help others by giving them the chance to have children. In contrast, the meaning of having a child of one’s own is valued so highly that the own genetic heritage is only linked to the own self. In our study, many participants stated that they would feel responsible for the child and would want to know about its well-being. This finding is in contrast to a post-donation study reporting that the oocyte donors felt no sense of responsibility towards the off- spring [21]. It has been shown that known donors tend to treat the child as any other child [17]. The absence of a genetic link has no negative impact on the well-being of the mother, father, or children at the age of three: An interview study showed even better interactions between parents and children in families after donor conception than in families with a naturally conceived child, implying the normal psychological development of the child [29].

Interestingly, the main concern of participants about gamete donation pertains to the genetic link. The majority of comments in the additional qualitative response section of our questionnaire refer to the meaning of genetic heritage and the well-being of the offspring. In our opinion, this issue shows that the population in Austria is not yet very familiar with gamete donation. Ethical concerns that play a minor role in countries with established gamete donation arise in our setting. We consider the concerns in our study to be fundamental, as they seem to question the meaning of gamete donation itself. In particular, women regard their oocytes “as part of myself” and “something very personal” and consider this child “to be my child forever”. Most comments do not take a limited view of the oocyte as a means to an end but as the basis for a child who needs its own biological mother to take care of him/her.

A post-donation study of gamete donors showed that female donors are more interested in the outcome of their donation and more altruistically motivated [20]. Interestingly, we found that more men than women were guided by altruistic motives.

### Limitations and suggestions for future research

The study focused on students at three Austrian universities, a non-representative study sample. We decided for a student population since the socio-demographic characteristics (education, age, relationship status, sexuality) of this cohort are comparable to donor features examined in other studies (e.g. [1, 7, 16, 30, 31]. Furthermore it is important to underline that the Austrian law on artificial procreation only allows women between 18 and 30 years to donate their oocytes for the sake of reproductive medicine. Male donors do not face this legal restriction.

Additionally, the majority of the study population was medical students. We assume that the topic of this study generated interest among these students because it is closely related to their field of study.

The main goal of the study was to analyse the willingness to donate gametes. As men and women can only donate their own gametes, we decided to compare sperm and oocyte donation by asking gender-specific questions about oocyte donation and sperm donation. We are well aware that the health consequences, time aspects, and effort related to the procedure of gamete donation present constraints in terms of comparability. However, as we lack empirical studies addressing the differences between women and men in regard to the willingness to donate gametes, it was a central concern of our study to scrutinize this comparison.

## Conclusion

In contrast to most previous studies in the field of gamete donation, our study has a pre-donation focus and concentrates on male and female donors. Hence, we are able to provide empirical findings about the willingness to donate sperm and oocytes and to elucidate influential attitudes and motives for oocyte and sperm donation in a population of potential donors. These findings contribute to a better understanding of potential donors’ perspectives within the existing donation framework and thereby help to evaluate the given regimes. Moreover, the type of evidence we provide may also support the design of improved donation frameworks.
